# Analysis of the Deleterious Single-Nucleotide Polymorphisms Associated With Antidepressant Efficacy in Major Depressive Disorder

**DOI:** 10.3389/fpsyt.2020.00151

**Published:** 2020-03-18

**Authors:** Juncai Xin, Meng Yuan, Yonglin Peng, Ju Wang

**Affiliations:** School of Biomedical Engineering, Tianjin Medical University, Tianjin, China

**Keywords:** major depressive disorder, antidepressants, non-synonymous SNPs, protein structure, molecular dynamics simulations

## Abstract

Major depressive disorder (MDD) is a serious mental disease with negative effects on both mental and physical health of the patient. Currently, antidepressants are among the major ways to ease or treat MDD. However, the existing antidepressants have limited efficacy in treating MDD, with a large fraction of patients either responding inadequately or differently to antidepressants during the treatment. Pharmacogenetics studies have found that the genetic features of some genes are associated with the antidepressant efficacy. In order to obtain a better understanding on the relationship between the genetic factors and antidepressant treatment response, we compiled a list of 233 single-nucleotide polymorphisms (SNPs) significantly associated with the antidepressant efficacy in treating MDD. Of the 13 non-synonymous SNPs in the list, three (rs1065852, rs3810651, and rs117986340) may influence the structures and function of the corresponding proteins. Besides, the influence of rs1065852 on the structure of CYP2D6 was further investigated via molecular dynamics simulations. Our results showed that compared to the native CYP2D6 the flexibility of the F-G loop was reduced in the mutant. As a portion of the substrate access channel, the lower flexibility of F-G loop may reduce the ability of the substrates to enter the channel, which may be the reason for the lower enzyme activity of mutant. This study may help us to understand the impact of genetic variation on antidepressant efficacy and provide clues for developing new antidepressants.

## Introduction

Major depressive disorder (MDD) is a serious mental disorder that influences ~6% population worldwide (1, 2). The lifetime incidence of depression is ~16.6% (3, 4), and the rate for females is twice that of males (5). The symptoms of MDD are complicated, including anxiety, cognitive impairment, suicidal tendencies, and emotional, somatic, and functional impairments (6). The disease can negatively affect almost all aspects of a person, including personal life, work life, and education, as well as general health. It does not only severely limit the psychosocial functioning and deteriorate life quality of the patients, but also brings heavy spiritual and economic burden to their families and the society (7). Actually, MDD is among the most burdensome disease worldwide (8, 9), both in the developed and developing countries (1, 10, 11), and the World Health Organization predicted that MDD will become the major cause of years lost to disability in 2030 (12). Thus, it is still a huge challenge to develop more effective therapeutic approaches that can accurately diagnose and treat MDD.

Currently, antidepressants are among the major ways to treat or alleviate MDD. The available antidepressants can be classified into several types according to their structure and the way they work, including selective serotonin reuptake inhibitors (SSRIs), monoamine oxidase inhibitors, serotonin and norepinephrine reuptake inhibitors, tricyclic antidepressants, and the other antidepressants. However, studies have shown that the existing antidepressants have limited efficacy in treating MDD, and the response of patients to antidepressants is difficult to predict (13). For example, it is found that the common antidepressants are effective in approximately only 40% of MDD patients (14), and 30–50% of patients have no response to the initial treatment of antidepressants (14). Even if a patient responds to an antidepressant, the recurrence rate is usually high (15).

The biological mechanisms of antidepressant response are still unclear, but earlier studies indicate that genetic factors may play an important part in this procedure (16–18). Pharmacogenetic studies show that the genetic characteristics of an individual can make an antidepressant more or less effective, and the single-nucleotide polymorphisms (SNPs) of some genes are related to the drug efficacy (19). Genes affecting antidepressant efficacy can be roughly grouped into two major categories, that is, genes of cytochrome P450 (CYP) family and those involved in the serotonin biochemical pathway (20). Members of the CYP family, for example, *CYP2D6, CYP1A2, CYP3A4*, and *CYP2C19*, have been found to play important roles in the metabolism of antidepressants (19, 21–23). Because an SNP may lead to abolished, reduced, or increased enzyme activity, some members of CYP families, such as CYP2D6, may have four phenotypes in individuals, including normal metabolizers, intermediate metabolizers, poor metabolizers, and ultrarapid metabolizers (24). Hence, there is a connection between the *CYP2D6* polymorphisms and the plasma levels of antidepressants; and MDD patients need a dose of antidepressant appropriate to their genetic characteristics to achieve better efficacy and fewer side effects. For genes in the serotonin biochemical pathway, such as the serotonin transporter gene (*SLC6A4*) and 5-hydroxytryptamine receptor 2A (*HTR2A*), genotypes are also associated with the antidepressant efficacy (19).

Until now, the association between genotype and the efficacy of antidepressants has been detected in a number of studies (25–27). For example, it is found that rs6265 in brain-derived neurotrophic factor (*BDNF*) is significantly associated with response to antidepressants in MDD (28–31). Some studies show that the SNPs in 5-hydroxytryptamine receptor 1B (*HTR1B*), *HTR1A*, and *HTR2A* are significantly associated with efficacy of antidepressants (32–34). Several genome-wide association studies (GWASs) have also been performed to detect the association between SNPs and antidepressant efficacy, such as the Pharmacogenomic Research Network Antidepressant Medication Pharmacogenomic Study (PGRN-AMPS) (35), the International SSRI Pharmacogenomics Consortium (ISPC) (36), the Sequenced Treatment Alternatives to Relieve Depression (STAR^*^D) study (37), the Genome-Based Therapeutic Drugs for Depression (GENDEP) project in whole sample (38), and the Munich Antidepressant Response Signature (MARS) (39). Although none of these studies reported the results that achieved the genome-wide significance threshold (40), a meta-analysis based on the data of PGRN-AMPS, ISPC, and STAR^*^D study identified an SNP (rs2456568) on hypoxanthine phosphoribosyltransferase pseudogene 4 (*HPRTP4*) at the genome-wide significance level (36). In another GWAS of antidepressant response in Koreans, two significant SNPs (rs7785360 and rs12698828) on autism susceptibility candidate 2 (*AUTS2*) were found to be related to antidepressant response (41).

Although a number of SNPs have been found to be significantly associated with antidepressant efficacy in treating MDD, it is still unclear how these SNPs affect the interaction between proteins and the drugs, as well as the response of antidepressants. Hence, exploring the correlation between the SNPs and the structures of the corresponding proteins may provide valuable information to understand the molecular mechanism underlying response to antidepressants and help us to develop novel antidepressants. In this article, we implemented a comprehensive curation of SNPs related with antidepressant efficacy from genetic studies and identified the non-synonymous SNPs (nsSNPs) potentially influencing the biological function and structure of the proteins.

## Materials and Methods

### SNPs Associated With of Antidepressant Efficacy for MDD

**Figure 1:**
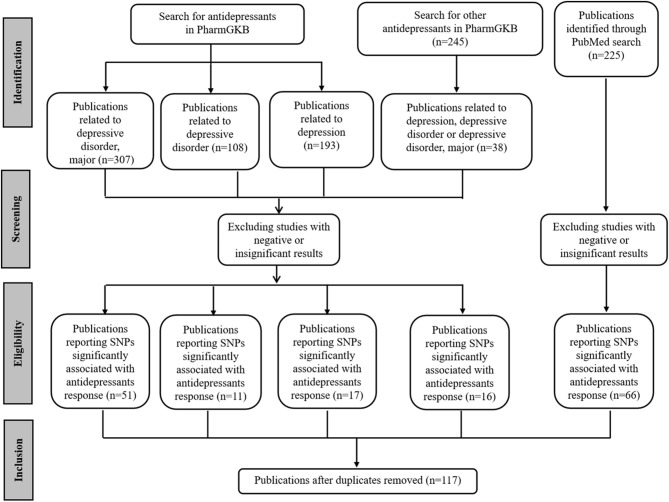
PRISMA flow diagram illustrating search strategy and studies included in the analysis. PRISMA, Preferred Reporting Items for Systematic Reviews and Meta-Analyses (http://www.prisma-statement.org/).

First, SNPs associated with antidepressant efficacy were collected. The Pharmacogenomics Knowledgebase (PharmGKB, https://www.pharmgkb.org/), a comprehensive resource for clinical information, gene-drug associations, and genotype-phenotype relationships (42), was queried with different keywords. For terms “antidepressants and depressive disorder, major,” “antidepressants and depressive disorder,” and “antidepressants and depression,” 307, 108, and 193 publications were retrieved respectively. In addition, another list of 245 publications related to other antidepressants was retrieved, among which the contents of 38 publications were closely related to depression. In addition, 225 publications were retrieved from PubMed (https://www.ncbi.nlm.nih.gov/pubmed/) via the query term “(Depressive Disorder, Major [Mesh]) AND (Antidepressive Agents [Mesh]) AND (Polymorphism, Single Nucleotide [Mesh] OR Pharmacogenetics [Mesh]).” For these publications, the redundant ones were removed. Then, the abstracts of the remaining publications were reviewed, and only studies related to antidepressant response were selected. Most of these studies were GWASs, meta-analysis, and candidate gene analysis of antidepressant responses. In each case, we selected only the SNPs that were significantly associated with the response of antidepressants in MDD. To reduce false-positive findings, the publications reporting negative or insignificant correlations were excluded, although some SNPs explored in these studies might be really associated with antidepressant responses. The full texts of the selected publications were examined to ensure the conclusions were consistent with the content. We narrowed our selection by focusing on those purporting one or more SNPs significantly associated with antidepressant responses. Finally, we retrieved 117 publications (Figure 1).

### Functional Analysis of nsSNPs

We estimated the influence of amino acid substitution on protein function by several tools, including SIFT (https://sift.bii.a-star.edu.sg/index.html), PolyPhen-2 (http://genetics.bwh.harvard.edu/pph2/), and SNAP2 (https://rostlab.org/services/snap2web/). SIFT is a tool to predict the change of protein function caused by single amino acid mutation based on homologous protein sequence and the physical properties of amino acids (43). The output of the tool is a tolerance index score measuring whether an amino acid substitution in a protein is tolerated or damaging, and an SNP with tolerance index score < 0.05 is defined to be deleterious. PolyPhen-2 predicts the effects of human nsSNPs on protein structure and function based on the features of sequence and structural information (44). The output of PolyPhen-2 is a score ranging from 0 to 1, with larger scores indicating higher likelihood of being damaging. SNAP2 is used to identify the potential effect of mutations by analyzing various sequence and variant features via the artificial neural network. It measures each substitution with a score ranging from −100 (strongly predicted “neutral”) to +100 (strongly predicted “effect”) (45). SNAP2 is used to differentiate between effect and neutral nsSNPs by thinking about the various sequence and variant features (45). And the score of each substitution ranges from −100 (strongly predicted “neutral”) to +100 (strongly predicted “effect”).

The mutant protein stability changes were analyzed by I-MUTANT 3.0 (http://gpcr2.biocomp.unibo.it/cgi/predictors/I-Mutant3.0/I-Mutant3.0.cgi). I-MUTANT can detect the changes of protein stability according to single amino acid substitution (46).

Because an evolutionary conservative an amino acid may be closely related to the protein structure and function, we assessed the conservation of amino acids in a given protein to identify the amino acids whose substitutions may be important for the molecule. The ConSurf (http://consurf.tau.ac.il/2016/) was utilized to evaluate the evolutionary conservation of amino acid positions in protein based on the phylogenetic relationships between homologous protein sequences (47).

### Analyzing the Structural Fluctuation Due to Deleterious nsSNPs by Molecular Dynamics Simulation

For purpose of detecting the influence of the most deleterious nsSNP on structure, we performed molecular modeling and molecular dynamics (MD) simulation for the native and mutant proteins, respectively. We selected the most closely related protein structure from PDB (Protein Data Bank, https://www.rcsb.org). Then, the structure of mutant protein was constructed based on the structure of native protein by SWISS-MODEL (https://swissmodel.expasy.org) (48). Finally, energy minimization and MD simulations were implemented on the native and mutant protein to investigate the structural deviation and fluctuation due to deleterious nsSNPs. All the analyses were implemented by using GROMACS 5.1.4 package (49).

We used the AMBER99SB-ILDN force field in both native and mutant model systems (50). The proteins being simulated were at the center of the cubic box. Then, we filled the box with the three-site transferrable intermolecular potential water solvent model and added chlorine ions to the system to neutralize counter-ions. The cutoff for short-range electrostatic and van der Waals interactions was selected as 1.4 nm. In the analysis, we first performed the energy minimizations for the initial structures by using the algorithm of the steepest descent minimization. Next, we conducted the equilibration via a two-stage procedure, that is, an NVT (*N*, constant number; *V*, volume; and *T*, temperature) ensemble was first conducted for 1.0 ns and stabilized the temperature of the system at 300 K; then, an NPT (*N*, constant number; *P*, pressure; and *T*, temperature) ensemble was conducted for 5.0 ns to ensure the system was well-equilibrated with respect to pressure and density. After the system is well-equilibrated at the expected temperature and pressure, we performed the MD simulations for 200 ns to explore the structural deviation and fluctuation of native and mutant protein due to deleterious nsSNPs. Finally, in order to evaluate the convergence of the MD simulations, we calculated the root-mean-square deviation (RMSD). Furthermore, root-mean-square fluctuation (RMSF) was calculated to assess the differences in structural flexibility between the native and mutant proteins. The RMSD and RMSF were obtained from the trajectory files of the MD results, which were produced after the MD simulation.

## Results

### SNPs Associated With of Antidepressant Efficacy for MDD

**Figure 2:**
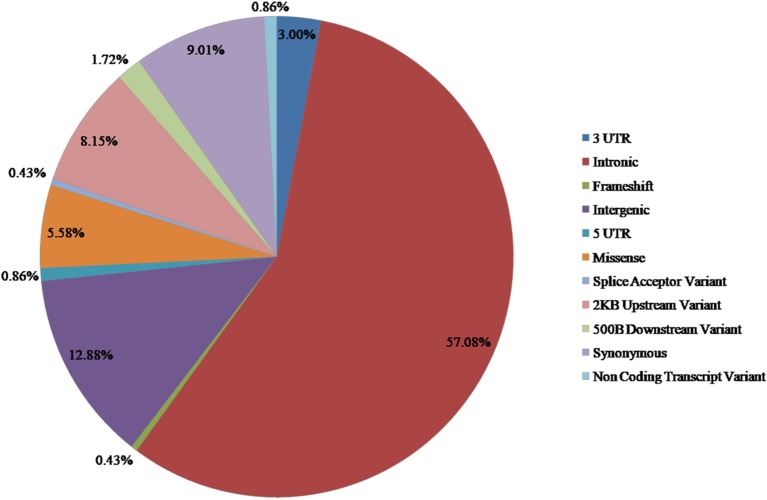
Distribution of SNPs associated with antidepressant response in MDD.

From the available studies, we collected 233 SNPs significantly associated with the antidepressant response (Supplementary Table 1). Of these SNPs, 13 were nsSNPs (missense), and 21 were synonymous SNPs in coding region. In the non-coding region, 133 SNPs were intron variant (intronic), and 7 and 2 SNPs were in 3′ UTR and 5′ UTR, respectively (Figure 2). These SNPs were mapped to 110 genes. Among them there were four serotonin receptors, that is, *HTR1A, HTR1B, HTR2A*, and *HTR7*; one dopamine receptor, that is, *DRD3*; two corticotropin-releasing hormone receptors, that is, *CRHR1* and *CRHR2*; two glutamate receptors, that is, *GRIK2* and *GRIK4*; two serotonin biosynthesis genes, that is, *TPH1* and *TPH2*; and some genes encoding transporters, that is, *SLC6A1, SLC6A2, SLC6A3, SLC6A4, SLC39A14*, and *ABCA1*. In addition, some genes were related to drug transport, that is, *CACNA1A, DTNBP1, GDNF, CRH, SLC6A2, SLC6A3, GAL, SNCA*, and *CNR1*; some genes were involved in the exogenous drug catabolic process, that is, *CYP1A2, CYP2C19*, and *CYP2D6*; and some genes were related to response to drug (*COMT, SRP19, NCAM1, NR3C1, CREB1, CRH, CRHBP, CRHR1, BDNF, GSK3B, ITPR2, ARRB2, SERPINE1, RGS17, MAPK1, SNCA*, and *IL1B*). The functional diversity of these genes clearly indicated the complexity of the mechanisms for antidepressant response. In addition, *ABCB1, TPH2, ANO2, ZNF385D*, and *CYP1A2* appeared more frequently in these genes. For these SNPs, most were located in the non-coding regions. Currently, it still remains a significant challenge to explain how SNPs located in intronic or intergenic regions affect drug response. In addition, because synonymous SNPs in the coding region do not change the protein sequence, it is difficult to experimentally address the function of every mutation seen in these regions. Therefore, we focused only on the nsSNPs in the coding region in the following analyses (Table 1).

**Table 1:** The nsSNPs associated with antidepressant response in MDD.

**SNP ID**	**Gene**	**Accession**	**Alleles**	**AA change**
rs2032582	ABCB1	NP_000918.2	TCT ⇒ GCT	S 893 A
rs6314	HTR2A	NP_000612.1	CAT ⇒ TAT	H 452 Y
rs6265	BDNF	NP_001137277.1	GTG ⇒ ATG	V 66 M
rs1065852	CYP2D6	NP_000097.3	CCA ⇒ TCA	P 34 S
rs2228479	MC1R	NP_002377.4	GTG ⇒ ATG	V 92 M
rs11580409	ERICH3	NP_001002912.4	TTA ⇒ GTA	L 1056 V
rs6280	DRD3	NP_000787.2	GGC ⇒ AGC	G 9 S
rs12603700	MIEF2	NP_683684.2	GGG ⇒ GAG	G 335 E
rs3889402	MIEF2	NP_001138372.1	GCA ⇒ TCA	A 204 S
rs3810651	GABRQ	NP_061028.3	ATT ⇒ TTT	I 478 F
rs4680	COMT	NP_000745.1	GTG ⇒ ATG	V 158 M
rs117986340	KMT2E	NP_061152.3	GGT ⇒ TGT	G 999 C
rs2072446	p75NTR	NP_002498.1	TCG ⇒ TTG	S 205 L

### Functional Analysis of nsSNPs

To explore the potential influence of amino acid substitutions on the function of protein caused by nsSNPs, we analyzed the 13 nsSNPs via SIFT, PolyPhen, and SNAP2. Of these nsSNPs, each tool predicted 5 as deleterious (Table 2), among which 3 nsSNPs (rs1065852, rs3810651, and rs117986340) were commonly identified by SIFT, PolyPhen-2, and SNAP2, which were selected for further investigation. Then, we performed I-MUTANT analysis on the three nsSNPs (Table 3). Because the DDG values for all these SNPs were negative (i.e., smaller than 0 kcal/mol), the amino acid substitutions might decrease the stability of the corresponding proteins.

**Table 2:** The functional analysis of nsSNPs using SIFT, PolyPhen-2, and SNAP2.

		SIFT		PolyPhen-2		SNAP2		
SNP ID	AA change	Score	Predicted effect	Score	Predicted effect	Score	Predicted effect	Expected accuracy%
rs2032582	S893A	1.00	Tolerated	0.000	Benign	−50	Neutral	72
rs6314	H452Y	0.02	Affect protein function	0.010	Benign	−11	Neutral	57
rs6265	V66M	0.18	Tolerated	0.822	Possibly damaging	−41	Neutral	72
rs1065852	P34S	0.00	Affect protein function	0.946	Possibly damaging	66	Effect	80
rs2228479	V92M	0.28	Tolerated	0.015	Benign	58	Effect	75
rs11580409	L1056V	1.00	Tolerated	0	Benign	−78	Neutral	87
rs6280	G9S	0.24	Tolerated	0	Benign	−28	Neutral	61
rs12603700	G335E	0.07	Tolerated	0.844	Possibly damaging	0	Neutral	53
rs3889402	A204S	0.00	Affect protein function	0	Benign	−13	Neutral	57
rs3810651	I478F	0.00	Affect protein function	0.662	Possibly damaging	67	Effect	80
rs4680	V158M	0.09	Tolerated	0.016	Benign	−8	Neutral	53
rs117986340	G999C	0.00	Affect protein function	1.000	Probably damaging	5	Effect	53
rs2072446	S205L	0.11	Tolerated	0.008	Benign	10	Effect	59

**Table 3:** Protein stability changes after amino acid substitution for nsSNPs predicted by I-MUTANT.

Gene	SNP ID	AA change	I-MUTANT Suite 3.0		
			SVM2 (kcal/mol)		
			DDG^a^ value prediction	Prediction effect	RI^b^
CYP2D6	rs1065852	P34S	−1.21	Decrease	8
GABRQ	rs3810651	I478F	−1.68	Decrease	9
KMT2E	rs117986340	G999C	−0.89	Decrease	4

a Free energy change value.

b *Reliability Index*.

As we know, the functional important domains of protein usually are highly conserved, which means if an nsSNP is located in conservative domains, it may potentially be important for maintaining the protein structure and function. The conservation of the three deleterious nsSNPs was analyzed using ConSurf. In this study, we performed ConSurf analysis through PSI-BLAST algorithm with default parameters to collect the homologous sequences in UniRef90 protein databases (47) (Table 4). The conservation scores of I478F (rs3810651) and G999C (rs117986340) were 1 and 3, respectively, revealing they were variable. But the conservation score of P34S (rs1065852) was 9, which indicated that the rs1065852 located in a conserved region and may be important for maintaining protein function.

**Table 4:** The evolutionary conservation analysis of nsSNPs by ConSurf.

**Gene**	**SNP ID**	**AA change**	**ConSurf**
			**UNIREF90**
CYP2D6	rs1065852	P34S	9
GABRQ	rs3810651	I478F	1
KMT2E	rs117986340	G999C	3

### Analyzing the Structural Fluctuation Due to Deleterious rs1065852 by Molecular Dynamics Simulation

**Figure 3:**
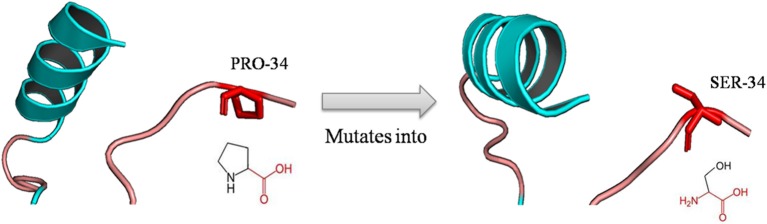
Schematic structures of the native (left) and mutant (right) amino acid. The backbone colored red and the same for each amino acid, and side chain unique for each amino acid is colored black; the mutation of proline into serine at position 34 for rs1065852.

The mutation of a proline (P) into a serine (S) occurred at position 34 due to deleterious nsSNPs rs1065852 (Figure 3). The difference (amino acid properties, structure) of proline (P) and serine (S) may lead to structural deviation and fluctuation, which may cause the functional deviations between the native and mutant CYP2D6. Thus, we implemented MD simulations for the native and mutant CYP2D6 structures to determine the reason of the structural difference.

The native structure of CYP2D6 was obtained from PDB (PDB ID: 3QM4) (51). Then, we deleted the prinomastat and waters from 3QM4 to construct the initial structure. Then, using 3QM4 as template, the initial structure of mutant was constructed by SWISS-MODEL. After the systems reached being well-equilibrated, 200-ns MD simulations were implemented for the initial structures of native and mutant CYP2D6.

**Figure 4:**
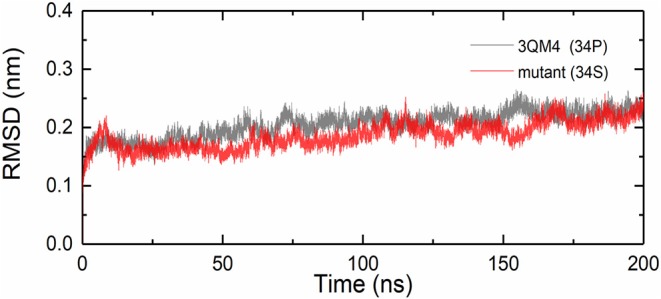
Root mean square deviations (RMSDs) for the native (black) and mutant (red) of CYP2D6.

We analyzed the RMSDs for backbone of the native and mutant CYP2D6 to evaluate the convergence of the systems. Root-mean-square deviation indicates the average change in displacement of the selected atoms for a particular frame in respect of a reference coordinate system. The initial structures were used as the references of RMSDs. Figure 4 shows that the simulations of native and mutant systems were both converged. In the last 20 ns of simulation, the average RMSD of native structure was 0.228 nm, whereas that of mutant structure was 0.219 nm. In both structures, the structural deviations were observed during simulations. In addition, the fluctuations of RMSDs were smaller at the end of the simulations, which indicated that the simulations generated stable trajectories. Therefore, the results could be used for further analysis.

**Figure 5:**
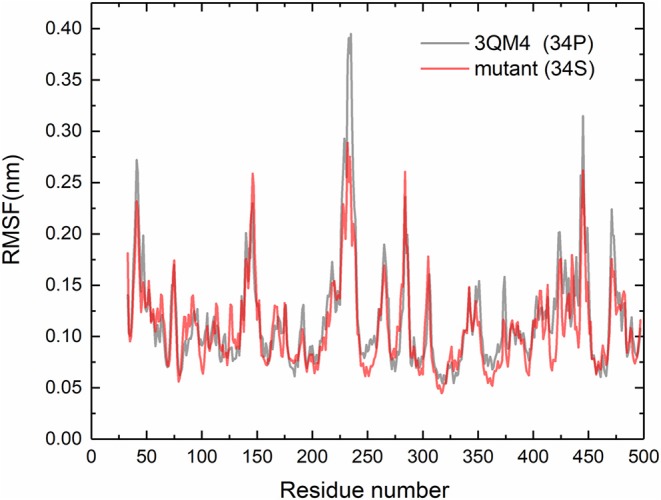
Root mean square fluctuations (RMSFs) for the native (gray) and mutant (red) of CYP2D6.

**Figure 6:**
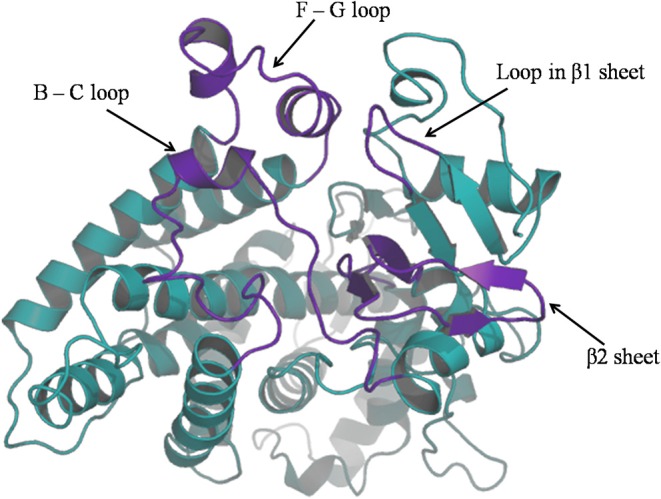
The ribbon diagram of CYP2D6; channel entrance is highlighted in purple.

For purpose of detecting the changes of flexibility between the native and mutant CYP2D6 structures, we calculated the RMSFs. The results are shown in Figure 5. For the RMSFs of native structure, the highest peak was observed near the 230th residue, that is, F-G loop in 3QM4, which indicated that this portion of the native CYP2D6 was flexible. While the RMSFs of mutant in this portion were lower, the flexibility of the F-G loop is reduced compared to the native CYP2D6. In addition, the entrance of the substrate access channel of CYP2D6 is made up of F-G loop, B-C loop, and the loop in the β1 sheet and β2 sheet (Figure 6). The CYP2D6 structural flexibility contributes to its catalytic versatility. As a constituent portion of the substrate access channel, the lower flexibility of F-G loop may affect the substrates that enter into the channel entrance. Hence, this may be the reason for the low enzyme activity of mutant.

## Discussion

Although a number of SNPs are found to be significantly associated with the response of antidepressants in MDD, it is still unclear how they affect the interaction between genes carrying these SNPs and antidepressants. In this study, we collected the SNPs significantly associated with the response of antidepressants and explored the potential mechanism underlying the different antidepressant efficacy among MDD patients. Most of the SNPs were included in the intronic or intergenic regions. For the 13 nsSNPs, three of them (rs1065852, rs3810651, and rs117986340) were predicted to be deleterious to the corresponding proteins. Evolutionary conservation analysis showed that P34S (rs1065852) was the only one located in the conserved protein domain, indicating it may be important for maintaining protein function. Subsequently, the results of the MD simulations revealed that the flexibility of the F-G loop for mutant was reduced compared to the native CYP2D6. As a constituent portion of the substrate access channel, this may affect the substrates that enter into the channel entrance. Hence, this may be the reason for the low enzyme activity of mutant.

In humans, there are ~60 CYP genes (52). The CYP enzymes are related to the synthesis and metabolism of various molecules and chemicals. As a member of the CYPs superfamily, CYP2D6 plays an important role in drug metabolism. It is responsible for the removal of at least 20% of the compounds, including antidepressants (53). Earlier studies have found the polymorphisms of CYP2D6 can cause the differences for antidepressant response among MDD patients, among which include CYP2D6 P34S (rs1065852). However, our understanding on the effect of the polymorphisms in CYP2D6 on the antidepressant efficacy is limited (54), and available studies have found mixed results. Some studies reported negative findings (55–57). For instance, Hodgson et al. (56) analyzed the data from GENDEP and found there was no significant association between CYP2D6 polymorphisms and response to antidepressants. In contrast, some studies reported positive associations between CYP2D6 polymorphisms and response to antidepressants (58–61). For example, Zastrozhin et al. (59) found that patients with 1846G>A of CYP2D6 polymorphism (rs3892097) had significantly reduced efficacy of fluvoxamine. For the P34S (rs1065852) substitution in CYP2D6. Tsai et al. (60) suggested that CYP2D6 polymorphisms (CYP2D6^*^4,CYP2D6^*^5, and CYP2D6^*^10) may be predicting patient treatment outcomes. In our study, we obtained the results of Han et al. (61), which found the P amino acid of the P34S (rs1065852) substitution in CYP2D6 is a favorable factor in the treatment of escitalopram for MDD and that the P34S (rs1065852) substitution may be a good genetic marker for predicting the treatment outcomes of escitalopram.

The results of the MD simulations indicated that the flexibility of the F-G loop for mutant was reduced compared to the native CYP2D6. The P34S(rs1065852) substitution is located in a highly conserved region encoding the proline-rich N-terminus in the three-dimensional structure of CYP2D6 (51), which was consistent with the results of our previous evolutionary conservation analysis. In addition, we analyzed the difference between the native and mutant amino acids in terms of amino acid properties, structure, and domains by HOPE (http://www.cmbi.ru.nl/hope/), which is a tool that analyzes the structural effects of a point mutation in protein sequence (62). For amino acid properties, the serine is smaller than the native residue proline at position 34, which may cause a loss of external interactions (62). And the native residue is more hydrophobic than the mutant residue (62), which might cause the damage of hydrophobic interactions with other molecules on the surface of CYP2D6. From the comparison of the structures of mutant and original amino acids, prolines are very rigid and produce a particular backbone conformation, which might be needed at this position. The amino acid substitution can disturb this particular conformation. For domain, the mutant residue may affect the domain that is important for the activity of CYP2D6 (62). Therefore, structural perturbation of this region may cause the change of protein stability and enzyme activity. Research showed that the P34S (rs1065852) affected the hydrogen bonding network in the interdomain between of the N-terminal and the F-G loop (63, 64). The results indicated that the hydrogen bonds located in the interdomain between the N-terminal and the F-G loop in mutant were fewer than in native CYP2D6. And the hydrophobic effect may alter the structure of F-G loop. Hence, the change of N-terminal loop due to the mutant P34S (rs1065852) influenced the hydrogen bonding network and ultimately may alter the structure of F-G loop. The mutation might disturb signal transfer from N-terminal to the distant protein from the mutated position through hydrophobic effect (64) and influence the structure of the distant residues from the mutant position. Other studies also reported the activity of mutant CYP2D6 has significantly reduced *in vivo* (65–67). The results of an early study suggested that the mutant of P34S affected the enzymatic activities (68). Kim et al. (69) indicated that the loss of functions in CYP2D6 alleles can be attributed to the P34S(rs1065852) substitution. These reports are consistent with our findings. The findings may facilitate us to understand why the P amino acid of the P34S (rs1065852) substitution in CYP2D6 is a favorable factor than S amino acid in the treatment of escitalopram for MDD and provide theoretical basis for the development of new antidepressants and personalized medicine in MDD.

Although rs3810651 and rs117986340 are not located in highly conserved regions, their involvements in the antidepressant efficacy are clear. SNP rs3810651 (I478F) is located in the coding region of γ-aminobutyric acid type A receptor, subunit theta (*GABRQ*), whose genotype AA + AT (F) is significantly associated with increased response to venlafaxine in people with MDD as compared to genotype TT(I) (70). GABRQ is the site of action of a number of important pharmacologic agents (71). The mutated residue phenylalanine is bigger than the original residue isoleucine at position 478 for rs3810651, and the phenylalanine is located in a domain that is important for the activity of GABRQ. Therefore, the mutation might have influence on the activity of the extracellular ligand-gated ion channel (62). The SNP rs117986340 (G999C)is located on lysine methyltransferase 2E (*KMT2E*), whose genotype GG(G) is significantly associated with increased response to duloxetine in people with MDD as compared to genotype GT(C) (72). The original residue glycine at position 999 is smaller and more hydrophilic than the mutated residue cysteine, which might be necessary for the function of KMT2E. In view of structure, glycine is more flexible to generate the torsion angles compared with the mutant residue; the mutation will result in an inaccurate conformation and disturb the local structure (62).

Besides, of the 13 nsSNPs, several other SNPs are also frequently studied. It is found that rs6314 genotype AG is associated with improved response when treated with paroxetine in people with MDD as compared to genotypes AA + GG (73, 74). The *HTR2A* carrying rs6314 is related to postsynaptic serotonin signaling and is a target for many antidepressants (75). HTR2A involves the serotonin synthesis, release, reuptake, and mediation of SSRIs in human brain. Therefore, the polymorphisms of HTR2A are associated with the response of antidepressants in MDD (34, 76, 77). The variation may be associated with antidepressant response by affecting serotonin signaling cascades (73). The rs6314 is an SNP in the third exon of *HTR2A* gene and leads to the change between histidine (His) and tyrosine (Tyr) at position 452. In addition, rs6311, rs17288723, rs7997012, rs9316233, rs6313, and rs2770296 of this gene are also significantly associated with antidepressant efficacy in MDD. This may result in the changes in protein structure and affect the interaction between HTR2A and antidepressants. In addition, several earlier studies found that rs6265 genotypes in *BDNF* were significantly associated with the response to antidepressants in MDD (29–31). *BDNF* is related to the survival of existing neurons, as well as the development and differentiation of new neurons and synapses in the central nervous system (78). The rs6265(Val66Met) locates within the signal peptide; it may be important for the maturation of the protein (62). Mutation may cause the change in structure and function of BDNF and ultimately result in the difference of antidepressant efficacy in individuals.

Undeniably, there are some limitations in the current study. First, our results depend on the retrieved existing studies that purported the SNPs associated with the antidepressant efficacy. Given that identification of such genetic factors is an ongoing process, more SNPs and genes may be identified in the future. Second, because of the difference in methods and sample size of the available studies, it is possible that some SNPs with nominal or moderate association with the antidepressant efficacy are not included in our analysis, and some SNPs collected might be false positive; hence, in order to obtain a more comprehensive SNPs list and validate the results, further investigation with a larger sample size or different technical approaches is needed. Finally, although we combined different approaches to identify the deleterious nsSNPs that may influence the structure and function of proteins, some may still be missed. With the SNP data and analysis approaches becoming more comprehensive and accurate, these problems would be avoided.

## Conclusion

In this study, we conducted a computational analysis on the SNPs associated with antidepressant efficacy. From 233 SNPs collected from available human pharmacogenetic studies, we screened 13 nsSNPs and found three of them (i.e., rs1065852, rs3810651, and rs117986340) were likely to be deleterious in the encoding proteins, particularly P34S (rs1065852) in CYP2D6. Results of the subsequent MD simulation regarding rs1065852 indicated that the flexibility of the F-G loop for mutant was reduced compared to the native CYP2D6, which may be responsible for the decrease in enzyme activity by hindering the substrate recognition and access. In addition, our results may provide theoretical basis for the development of new antidepressants and personalized medicine in MDD.

## Data Availability Statement

All datasets generated for this study are included in the article/Supplementary Material.

## Author Contributions

JX, MY, YP, and JW: conceived, designed, and performed the experiments and wrote the paper. JX, MY, and YP: analyzed the data.

### Conflict of Interest

The authors declare that the research was conducted in the absence of any commercial or financial relationships that could be construed as a potential conflict of interest.
