# Supplementary material for: Analysis of the Deleterious Single-Nucleotide Polymorphisms Associated With Antidepressant Efficacy in Major Depressive Disorder
Source: Front Psychiatry. 2020 Mar 18;11:151. doi: 10.3389/fpsyt.2020.00151 (PMC7093583; doi:10.3389/fpsyt.2020.00151)
Supplement: Supplementary file 1 [file Table_1.DOC]

Supplemental Table S1. List of single nucleotide polymorphisms associated with

antidepressants response in MDD

| SNP ID | GENES | Localization | Reference^a^ |
| --- | --- | --- | --- |
| rs13306278 | COMT | 2KB Upstream Variant | [20877297](https://www.pharmgkb.org/literature/6324630) |
| rs1800544 | ADRA2A | 2KB Upstream Variant | 25642918  25710119  18794646 |
| rs3800373 | FKBP5 | 3 UTR | 15565110  23733030  18597649 |
| rs1360780 | FKBP5 | Intronic | 15565110  23733030  18597649  26645208 |
| rs41271330 | BMP5 | Synonymous | 23926243 |
| rs11042725 | ADM | 2KB Upstream Variant | 19636336 |
| rs4570625 | TPH2 | 2KB Upstream Variant | 27660918 |
| rs130058 | HTR1B | 5 UTR | 27660918 |
| rs10879346 | TPH2 | Intronic | 18496129 |
| rs2273623 | PPM1A | 3 UTR | 20970119 |
| rs4713916 | FKBP5 | Intronic | 18191112  15565110 |
| rs10975641 | GLDC | Intronic | 21107318 1107318 |
| rs1954787 | GRIK4 | Intronic | 17671280  19924111  25303296 |
| rs17060812 | SLC39A14 | Intronic | 20970119 |
| rs1800532 | TPH1 | Intronic | 23221997  21485747 |
| rs2032582 | ABCB1 | Missense | 24663076  17913323 |
| rs4646427 | CYP1A2 | Intronic | 21121774  23859573 |
| rs4646425 | CYP1A2 | Intronic | 21121774  23859573 |
| rs6314 | HTR2A | Missense | 18253134  11311507 |
| rs520210 | NEDD4L | Intronic | 23809733 |
| rs8136867 | MAPK1 | Intronic | 23537502 |
| rs28364032 | CRHR1 | 3 UTR | 24422887 |
| rs1449683 | FGF2 | Synonymous | 25710119 |
| rs495794 | SRP19 | Intronic | 22795047 |
| rs153549 | REEP5 | Intronic | 22795047 |
| rs7569963 | METTL21A | Intronic | 20643483 |
| rs153560 | REEP5 | Intronic | 22795047 |
| rs2500535 | UST | Intronic | 20360315  24151802 |
| rs5443 | GNB3 | Synonymous | 20826553  25451402  14647404  23733030 |
| rs1516338 | CHL1 | 2KB Upstream Variant | 25943212 |
| rs2216711 | GDNF | Intronic | 24577123 |
| rs2973049 | GDNF | Intronic | 24577123 |
| rs6265 | BDNF | Missense | 23619509  23733030  24577123  19236730  20674983  25658497  21934642  21485747 |
| rs2470890 | CYP1A2 | Synonymous | 21121774  23859573 |
| rs2472304 | CYP1A2 | Intronic | 21121774  23859573 |
| rs365836 | CUX1 | Intronic | 22584459 |
| rs1065852 | CYP2D6 | Missense | 24302953  24528284 |
| rs201522 | CUX1 | Intronic | 22584459 |
| rs6311 | HTR2A | 2KB Upstream Variant | 16874005 |
| rs352428 | EXTL3 | Intronic | 23324805 |
| rs2228478 | MC1R | Synonymous | 21052032 |
| rs2228479 | MC1R | Missense | 21052032 |
| rs242941 | CRHR1 | Intronic | 17258395 |
| rs57098334 | SLC6A4 | Intronic | 10683861  17018806 |
| rs889895 | CREB1 | Intronic | 23537502 |
| rs17288723 | HTR2A | Intronic | 19924111 |
| rs12800734 | GRIK4 | Intronic | 19924111 |
| rs7997012 | HTR2A | Intronic | 16642436  17671280  19924111  19077664  25108775  19758789 |
| rs7124442 | BDNF | 3 UTR | 19236730 |
| rs1126757 | IL11 | Synonymous | 23142150 |
| rs10997242 | CTNNA3 | Intronic | 27091189 |
| rs2299267 | PON2 | Intronic | 27091189 |
| rs2112460 | CACNA1A | Intronic | 27091189 |
| rs672170 | RGS17 | Intronic | 27091189 |
| rs766127 | MTRF1L | Intronic | 27091189 |
| rs9316233 | HTR2A | Intronic | 19365399 |
| rs2831440 | LINC01697 | intronic | 27091189 |
| rs852977 | NR3C1 | Intronic | 19365399 |
| rs4737771 | CRH (100 Kbp) | intergenic | 27091189 |
| rs49411 | FHIT | Intronic | 27091189 |
| rs6313 | HTR2A | Synonymous | 25108775 |
| rs12082710 | TGFBR3 | Intronic | 23092981 |
| rs11580409 | ERICH3 | Missense | 26903268 |
| rs6280 | DRD3 | Missense | 22926616 |
| rs7103411 | BDNF | Intronic | 19236730 |
| rs2227631 | SERPINE1 | 2KB Upstream Variant | 18794724 |
| rs1799889 | SERPINE1 | 2KB Upstream Variant | 18794724 |
| rs2270007 | CRHR2 | Intronic | 17467808 |
| rs1799752 | ACE | Intronic | 27262302 |
| rs56355515 | MIEF2 | 500B Downstream Variant | 29407288 |
| rs2032583 | ABCB1 | Intronic | 18215618  25847751 |
| rs7035619 | TEX10 | Intronic | 29407288 |
| rs7472 | TEX10 | Synonymous | 29407288 |
| rs12595802 | ATP10A | Intronic | 29407288 |
| rs12603700 | MIEF2 | Missense | 29407288 |
| rs3889402 | MIEF2 | Missense | 29407288 |
| rs10124893 | TEX10 | Intronic | 29407288 |
| rs10989064 | TEX10 | Intronic | 29407288 |
| rs6479008 | TEX10 | Intronic | 29407288 |
| rs3810651 | GABRQ | Missense | 23394390 |
| rs12502866 | LINC00504 | intronic | 29407288 |
| rs1487278 | TPH2 | Intronic | 18496129 |
| rs3761555 | GRIA3 | 2KB Upstream Variant | 23394390 |
| rs4261893 | ZNF385D | Intronic | 28696415 |
| rs502434 | GRIA3 | Synonymous | 23394390 |
| rs12657120 | TTC37 | Intronic | 29407288 |
| rs2242446 | SLC6A2 | Intronic | 28068779 |
| rs3761554 | GRIA3 | 2KB Upstream Variant | 23394390 |
| rs4148740 | ABCB1 | Intronic | 18215618 |
| rs6295 | HTR1A | 2KB Upstream Variant | 18484082  25710119  19326813  16302021  18387740 |
| rs61908402 | ANO2 | Intronic | 29407288 |
| rs10280101 | ABCB1 | Intronic | 18215618 |
| rs61908403 | ANO2 | Intronic | [29407288](https://www.pharmgkb.org/literature/15100160) |
| rs7787082 | ABCB1 | Intronic | [18215618](https://www.pharmgkb.org/literature/6387201) |
| rs61908404 | ANO2 | Intronic | [29407288](https://www.pharmgkb.org/literature/15100160) |
| rs4971678 | NRXN1 | Intronic | [29407288](https://www.pharmgkb.org/literature/15100160) |
| rs61908405 | ANO2 | Intronic | [29407288](https://www.pharmgkb.org/literature/15100160) |
| rs17724464 | ANO2 | Intronic | [29407288](https://www.pharmgkb.org/literature/15100160) |
| rs4148739 | ABCB1 | Intronic | [18215618](https://www.pharmgkb.org/literature/6387201) |
| rs17724494 | ANO2 | Intronic | [29407288](https://www.pharmgkb.org/literature/15100160) |
| rs11983225 | ABCB1 | Intronic | [18215618](https://www.pharmgkb.org/literature/6387201) |
| rs17786394 | ANO2 | Intronic | [29407288](https://www.pharmgkb.org/literature/15100160) |
| rs17786400 | ANO2 | Intronic | [29407288](https://www.pharmgkb.org/literature/15100160) |
| rs17786412 | ANO2 | Intronic | [29407288](https://www.pharmgkb.org/literature/15100160) |
| rs10771997 | LOC374443 | 500B Downstream Variant | [29407288](https://www.pharmgkb.org/literature/15100160) |
| rs10248420 | ABCB1 | Intronic | [18215618](https://www.pharmgkb.org/literature/6387201) |
| rs10771998 | LOC374443 | 500B Downstream Variant | [29407288](https://www.pharmgkb.org/literature/15100160) |
| rs2235040 | ABCB1 | Intronic | [18215618](https://www.pharmgkb.org/literature/6387201) |
| rs4680 | COMT | Missense | 23706899  20619611  18989660  22417933  24446536  17522626 |
| rs10771999 | LOC374443 | 500B Downstream Variant | [29407288](https://www.pharmgkb.org/literature/15100160) |
| rs12720067 | ABCB1 | Intronic | [18215618](https://www.pharmgkb.org/literature/6387201) |
| rs17724452 | ANO2 | Intronic | [29407288](https://www.pharmgkb.org/literature/15100160) |
| rs2303377 | NCAM1 | Intronic | [28696415](https://www.pharmgkb.org/literature/15099968) |
| rs4639250 | TTC37 | Intronic | [29407288](https://www.pharmgkb.org/literature/15100160) |
| rs9369266 | TREML4 | Intronic | [29407288](https://www.pharmgkb.org/literature/15100160) |
| rs1045642 | ABCB1 | Synonymous | [26664259](https://www.pharmgkb.org/literature/15096466) |
| rs117986340 | KMT2E | Missense | [28696415](https://www.pharmgkb.org/literature/15099968) |
| rs3124955 | FCN2 | Intronic | [29407288](https://www.pharmgkb.org/literature/15100160) |
| rs3128624 | FCN2 | Intronic | [29407288](https://www.pharmgkb.org/literature/15100160) |
| rs948854 | GAL | 2KB Upstream Variant | [20237460](https://www.pharmgkb.org/literature/15029593) |
| rs61692318 | LINC02267 | intronic | [29407288](https://www.pharmgkb.org/literature/15100160) |
| rs2235015 | ABCB1 | Intronic | 18215618  25847751 |
| rs9310658 | ZNF385D | Intronic | [29407288](https://www.pharmgkb.org/literature/15100160) |
| rs9819548 | ZNF385D | Intronic | [29407288](https://www.pharmgkb.org/literature/15100160) |
| rs9824595 | ZNF385D | Intronic | [29407288](https://www.pharmgkb.org/literature/15100160) |
| rs9873889 | ZNF385D | Intronic | [29407288](https://www.pharmgkb.org/literature/15100160) |
| rs9879065 | ZNF385D | Intronic | [29407288](https://www.pharmgkb.org/literature/15100160) |
| rs61908411 | ANO2 | Intronic | [29407288](https://www.pharmgkb.org/literature/15100160) |
| rs78482393 | ANO2 | Intronic | [29407288](https://www.pharmgkb.org/literature/15100160) |
| rs7616119 | ZNF385D | Intronic | [29407288](https://www.pharmgkb.org/literature/15100160) |
| rs78615940 | ANO2 | Intronic | [29407288](https://www.pharmgkb.org/literature/15100160) |
| rs7653345 | ZNF385D | Intronic | [29407288](https://www.pharmgkb.org/literature/15100160) |
| rs9310657 | ZNF385D | Intronic | [29407288](https://www.pharmgkb.org/literature/15100160) |
| rs61908406 | ANO2 | Intronic | [29407288](https://www.pharmgkb.org/literature/15100160) |
| rs61908407 | ANO2 | Intronic | [29407288](https://www.pharmgkb.org/literature/15100160) |
| rs12630569 | ZNF385D | Intronic | [29407288](https://www.pharmgkb.org/literature/15100160) |
| rs61908408 | ANO2 | Intronic | [29407288](https://www.pharmgkb.org/literature/15100160) |
| rs13093500 | ZNF385D | Intronic | [29407288](https://www.pharmgkb.org/literature/15100160) |
| rs2235067 | ABCB1 | Intronic | [18215618](https://www.pharmgkb.org/literature/6387201) |
| rs61908409 | ANO2 | Intronic | [29407288](https://www.pharmgkb.org/literature/15100160) |
| rs4334661 | ZNF385D | Intronic | [29407288](https://www.pharmgkb.org/literature/15100160) |
| rs61908410 | ANO2 | Intronic | [29407288](https://www.pharmgkb.org/literature/15100160) |
| rs116692768 | ITGA9 | Intronic | 29160301 |
| rs1049353 | CNR1 | Synonymous | 18579347 |
| rs41423247 | NR3C1 | Intronic | 28641498 |
| rs12760036 | NGF | Intronic | 26021968 |
| rs7523654 | NGF | Intronic | 26021968 |
| rs17033692 | NGF | Intronic | 26021968 |
| rs7963717 | TPH2 | 2KB Upstream Variant | 27521242 |
| rs2171363 | TPH2 | Intronic | 27521242  19272410 |
| rs4522461 | ARRB2 | Intronic | 29031912 |
| rs8076005 | SLC6A4 | Intronic | 24958631 |
| rs165737 | COMT | Intronic | 21788083 |
| rs10245483 | STEAP2-AS1 | Intronic | 25815420 |
| rs6296 | HTR1B | Synonymous | 21937687 |
| rs6298 | HTR1B | Synonymous | 21937687 |
| rs2072446 | NGFR | Missense | 18081157 |
| rs10473984 | CRHBP | Intronic | 20368512 |
| rs2770296 | HTR2A | Intronic | 22947179 |
| rs6347 | SLC6A3 | Synonymous | 22947179 |
| rs2289657 | NTRK2 | Synonymous | 19844206 |
| rs56142442 | NTRK2 | Synonymous | 19844206 |
| rs1083801 | GRM7 | Intronic | 22884879 |
| rs2005976 | DTNBP1 | Intronic | 17264804 |
| rs760761 | DTNBP1 | Intronic | 17264804 |
| rs2230372 | ITPR2 | Synonymous | 22119081 |
| rs2280272 | PRKCZ | Synonymous | 22119081 |
| rs17109671 | PLCE1 | Synonymous | 22119081 |
| rs17109674 | PLCE1 | Synonymous | 22119081 |
| rs25531 | SLC6A4 | 2KB Upstream Variant | 24446536 |
| rs16944 | IL1B | 2KB Upstream Variant | 20044070 |
| rs1143643 | IL1B | Intronic | 20044070 |
| rs10482633 | NR3C1 | Intronic | [19365399](https://www.pharmgkb.org/literature/2064161) |
| rs7305115 | TPH2 | Synonymous | 21937687 |
| rs16873129 | RAPGEF5(dist=12837) | Intergenic | 27091189 |
| rs521093 | EPS8L3(dist=61586) | Intergenic | 27091189 |
| rs2433320 | PDLIM5(dist=2203) | Intergenic | 24040476 |
| rs58042962 | SMCR7（80bp） | Intergenic | 29407288 |
| rs62319299 | C4orf33(dist=136379),  LINC02466(dist=471156) | Intergenic | 29407288 |
| rs10123866 | INVS | 3 UTR | 29407288 |
| rs2419128 | DPT(dist=12216) | Intergenic | 29407288 |
| rs4437856 | DPT(dist=13571) | Intergenic | 29407288 |
| rs6700741 | RN5S69（36kb） | Intergenic | [29407288](https://www.pharmgkb.org/literature/15100160) |
| rs2933304 | SLC6A1(dist=15924) | Intergenic | [29407288](https://www.pharmgkb.org/literature/15100160) |
| rs11933890 | C4orf33(dist=132791),  LINC02466(dist=474744) | Intergenic | [29407288](https://www.pharmgkb.org/literature/15100160) |
| rs1364043 | HTR1A(dist=5024) | Intergenic | 18484082  19326813 |
| rs55881666 | C4orf33(dist=138689),  LINC02466(dist=468846) | Intergenic | [29407288](https://www.pharmgkb.org/literature/15100160) |
| rs56229625 | C4orf33(dist=138688),  LINC02466(dist=468847) | Intergenic | [29407288](https://www.pharmgkb.org/literature/15100160) |
| rs10007051 | C4orf33(dist=127673),  LINC02466(dist=479862) | Intergenic | [29407288](https://www.pharmgkb.org/literature/15100160) |
| rs4858478 | UBE2E2-AS1(dist=62229) | Intergenic | [29407288](https://www.pharmgkb.org/literature/15100160) |
| rs7625956 | UBE2E2-AS1(dist=62683) | Intergenic | [29407288](https://www.pharmgkb.org/literature/15100160) |
| rs1908557 | GPRIN3(dist=192402),  SNCA(dist=223897) | Intergenic | [27622933](https://www.pharmgkb.org/literature/15098901) |
| rs1321744 | TBX18(dist=417428),  LINC02535(dist=205193) | Intergenic | 25220861 |
| rs2929115 | IDO2(dist=27887) | Intergenic | 22282879 |
| rs585719 | LINC01198(dist=15488),  LRCH1(dist=70897) | Intergenic | 23158458 |
| rs9361235 | HTR1B | Intronic | 28025020 |
| rs9361233 | HTR1B | Intronic | 28025020 |
| rs334558 | GSK3B | 2KB Upstream Variant | 18195729 |
| rs9315310 | NBEA(dist=164993) | Intergenic | 27091189 |
| rs2532560 | PARP11(dist=179257) | Intergenic | 27091189 |
| rs12094644 | DPT(dist=6467) | Intergenic | 29407288 |
| rs10042486 | HTR1A(dist=3210) | Intergenic | 18484082 19326813 |
| rs7306991 | CLEC2D | Non Coding Transcript Variant | [29407288](https://www.pharmgkb.org/literature/15100160) |
| rs7316769 | CLEC2D | Non Coding Transcript Variant | [29407288](https://www.pharmgkb.org/literature/15100160) |
| rs13204353 | TREML4 | 3 UTR | [29407288](https://www.pharmgkb.org/literature/15100160) |
| rs11178998 | TPH2 | 5 UTR | 27521242 |
| rs4790694 | ARRB2(dist=1559) | Intergenic | 29031912 |
| rs2929116 | IDO2(dist=26809) | Intergenic | 22282879 |
| rs76191705 | NRXN3 | Intronic | 29160301 |
| rs11179027 | TPH2 | Intronic | 25226239 |
| rs543196 | GRIK2 | Intronic | 25226239 |
| rs3828275 | GAD1 | Intronic | 25226239 |
| rs17110532 | TPH2 | Intronic | 25226239 |
| rs2066713 | SLC6A4 | Intronic | 25226239 |
| rs572487 | GRIK2 | Intronic | 25226239 |
| rs12185692 | GAD1 | Intronic | 25226239 |
| rs2020942 | SLC6A4 | Intronic | 25226239 |
| rs4760815 | TPH2 | Intronic | 25226239 |
| rs17110747 | TPH2 | 3 UTR | 25226239 |
| rs13321783 | GSK3B | Intronic | 18195729 |
| rs2319398 | GSK3B | Intronic | 18195729 |
| rs2075507 | COMT | 2KB Upstream Variant | 20531207 |
| rs10501087 | BDNF | Intronic | 21188787 |
| rs7905446 | HTR7 | 2KB Upstream Variant | 30874608 |
| rs762551 | CYP1A2 | Intronic | 23859573  21121774 |
| rs3743484 | CYP1A2 | Intronic | 23859573 |
| rs2069526 | CYP1A2 | Intronic | 23859573 |
| rs2069521 | CYP1A2(dist=2219) | Intergenic | 23859573 |
| rs5030655 | CYP2D6 | Frameshift | 16958828 |
| rs3892097 | CYP2D6 | Splice Acceptor Variant | 16642541 |
| rs1074145 | CYP2C19(dist=66542) | Intergenic | 24528284 |
| rs11188072 | CYP2C19(dist=3377) | Intergenic | 19884907 |
| rs12248560 | CYP2C19 | 2KB Upstream Variant | 19884907 |

^a^ The PubMed reference number PMID
